# Non‐Communicable Disease, Metabolic and Lifestyle Risk Factor Profiles in South African University Students: A Latent Class Analysis

**DOI:** 10.1002/puh2.70221

**Published:** 2026-04-09

**Authors:** Melissa Janse van Vuren, Wayne Derman, Jason Bantjes, Innocent Maposa, Liske Kotze‐Horstmann

**Affiliations:** ^1^ Division of Sport and Exercise Medicine Department of Exercise Sport and Lifestyle Medicine Faculty of Medicine and Health Sciences Stellenbosch University Cape Town South Africa; ^2^ South Africa IOC Research Centre Cape Town South Africa; ^3^ Rehabilitation Medicine Research Group Department of Health Sciences Lund University Sweden; ^4^ Mental health, Alcohol, Substance use and Tobacco Research Unit South African Medical Research Council Cape Town South Africa; ^5^ Department of Psychiatry and Mental Health University of Cape Town Cape Town South Africa; ^6^ Department of Global Health, Institute for Life Course Health Research Stellenbosch University Cape Town South Africa; ^7^ Division of Epidemiology and Biostatistics Department of Global Health Faculty of Medicine and Health Sciences Stellenbosch University Cape Town South Africa; ^8^ Division of Epidemiology & Biostatistics School of Public Health Faculty of Health Sciences Witwatersrand University Johannesburg South Africa

**Keywords:** common mental disorders, lifestyle, lifestyle intervention, metabolic risk factors, non‐communicable diseases, university students

## Abstract

**Background:**

Cardiovascular disease, chronic respiratory diseases, diabetes and cancer (referred to as NCD4), the four main groups of non‐communicable diseases (NCDs) and common mental disorders (CMDs) are key NCDs with shared risk factors, often established in youth. University students, known for their unhealthy lifestyles, represent a critical population for early intervention. However, few studies have examined their NCD risk profile.

**Aim:**

To assess self‐reported NCD4, CMDs, metabolic and lifestyle risk factor prevalence and the latent risk profiles present in a South African university student population.

**Methods:**

This cross‐sectional descriptive study used 2021–2023 data from the MaRooN Health Passport, an online health survey emailed to one university (*n* = 3252). Latent class analysis (LCA) identified naturally occurring subgroups based on NCDs, metabolic and lifestyle risk factor patterns.

**Results:**

Most participants had a median age of 21, were women (59.7%), undergraduates (76.5%) and attended non‐medical faculties (82.2%). The overall NCD prevalence was 52.5%. Chronic respiratory diseases (34.7%), CMDs (31.2%), raised body mass index (35.4%) and various lifestyle risk factors were prevalent. LCA identified three latent risk profiles. Although poor nutrition and alcohol use were common across groups, stress‐related factors (psychological distress, poor sleep and CMDs) differentiated the moderate‐ (28.7%) and higher risk (14.8%) groups from the lower risk group (56.5%), with increased substance use uniquely marking the higher risk group. NCDs and lifestyle risk factor prevalence and profiles differed according to gender.

**Conclusion:**

The characteristics of the latent risk profiles and NCD distribution identified in this study position university students as an ‘early‐risk’ population. Co‐occurring stress‐related factors and increased substance use characterised the higher risk group, whereas poor diet was common across profiles. Despite limitations, these findings highlight specific modifiable risk factors that can be addressed in this low‐resource setting by combining population‐level nutrition strategies with targeted, multi‐behavioural interventions focusing on stress‐related factors and substance use to improve student health.

## Graphical abstract

The NCD prevalence and overall risk profile of South African university students are understudied, despite widespread unhealthy lifestyles. Chronic respiratory diseases, CMDs and lifestyle risk factors are common. Findings support targeted interventions addressing stress‐related factors and substance use, alongside population‐level strategies to improve factors, such as poor diet.

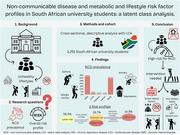

## 1 Introduction

University students represent a key population for disease prevention, as this transitional life phase is marked by increasing autonomy, experimentation and a challenging work‐life balance, resulting in the consolidation of often unhealthy lifestyle patterns that can track into adulthood [1, 2, 3]. Universities are uniquely positioned to create environments, policies and cultures that promote student health and are recognised as critical settings for disease prevention within the 2015 Okanagan Charter for Health Promoting Universities and Colleges framework [4, 5, 6]. As relatively self‐contained settings, universities may offer one of the last opportunities to implement comprehensive, population‐level health interventions before young adults enter the workforce [2, 5]. As such, targeting student health during this period provides an opportunity to support their well‐being and mitigate the risk of future disease [7]. However, context‐specific research is needed to identify distinct priorities for campus health initiatives and policy development, as universities may have unique social, cultural and environmental conditions that influence student health and shape specific patterns of behaviour [7, 8].

In South Africa, high prevalence rates of lifestyle risk factors, such as poor nutrition, alcohol use, smoking, inadequate physical activity and sedentary behaviour, have been identified among university students [9, 10, 11]. These and metabolic risk factors, such as hypertension and dyslipidaemia, constitute some of the main drivers of non‐communicable disease (NCD) development [12]. NCDs are chronic, non‐infectious diseases with shared behavioural, metabolic and environmental risk factors [12]. NCDs are a major public health concern due to their contribution to global mortality rates and impact on quality of life, educational pursuits and overall functioning [13, 14, 15, 16]. In particular, cardiovascular diseases, chronic respiratory diseases, cancer and diabetes, collectively called NCD4, contribute the most to mortality and morbidity rates and are considered key intervention targets by the World Health Organisation (WHO) Global Action Plan [16, 17]. Because common mental disorders (CMDs), such as anxiety and depression, are highly associated with NCDs and share risk factors, the WHO has advocated an integrated approach to addressing both [18, 19, 20, 21]. It has also been suggested that CMDs be included under the umbrella term of NCDs [21, 22, 23]. However, this approach remains controversial due to concerns about increased costs, reduced intervention efficacy and the lack of consideration of factors, such as stigmatisation [21, 22]. As this article focuses on overlapping determinants, the term ‘NCD’ refers to both NCD4 and CMDs. In South Africa, limited evidence suggests that between 26.8% and 78.4% of young adults have at least one NCD [24, 25]. This wide range in NCD prevalence may be attributed to methodological differences across studies, including participant recruitment (national survey vs. targeted recruitment), data collection (in‐person vs. online) and the heterogeneity of NCDs and the age ranges investigated, among others [24, 25]. However, the specific NCD prevalence among South African university students, who constitute approximately 21% of South African 18 to 24‐year‐olds, is unknown [26].

To better understand NCDs and NCD‐associated risk factors among university students, advanced statistical techniques, such as latent class analysis (LCA), can be used to identify latent risk profiles based on variable clustering [27, 28]. LCA provides a person‐centred approach that identifies distinct subgroups of individuals who share similar patterns of behaviours/characteristics by examining the relationship between those characteristics and subgroup membership, rather than variable‐centred methods like regression analysis, which model relationships among variables across an entire sample [29]. Identifying the risk profiles among these participants may provide insight into their health determinants, inform campus health promotion and intervention policies and provide a strategic entry point for large‐scale NCD prevention within this low‐resource university setting.

Thus, this study aims to determine the NCD and NCD‐associated risk profiles of a sample of South African university students who completed the *Maties at‐Risk for Non‐communicable disease* (MaRooN) Health Passport during 2021–2023 by describing their lifetime prevalence of NCD4 and CMDs and their metabolic‐ and lifestyle risk factors and investigating the latent risk profile present based on the NCD and risk factor patterns observed and their associations with demographic variables.

## 2 Materials and Methods

### 2.1 Study Design, Setting and Sample Population

This retrospective cross‐sectional descriptive study used data from the MaRooN Health Passport (MHP), a longitudinal online surveillance tool designed to identify trends in common diseases and risk factors among university staff and students. All registered students were invited to complete the MHP via an email link sent periodically to their university email addresses. Participants were informed about the purpose of the MHP and asked to complete the survey and consent to the collection of their data. More information about the MHP can be found elsewhere [9]. All participants 18 years and older who reported their gender, completed the MHP between April 2021 and December 2023, were registered for any undergraduate or postgraduate programme and consented to using their de‐identified data were included. All data were self‐reported, and objective clinical verification was not available. Ethical approval for this study was obtained from the university's Health Research Ethics Committee (HREC Reference No: S24/02/047).

### 2.2 Data Collection

#### 2.2.1 Demographic Details

Demographic data, including age, gender and level of study (undergraduate or postgraduate), were extracted from the MHP database. Health science students may share similar lifestyle risk factors with non‐health science students, despite the health science field focusing on health and lifestyle education [30]. This has not been investigated in this cohort, and the health sciences faculty is located on a separate campus. Therefore, to assess whether students from these faculties/campuses could be combined, data were also extracted by medical and non‐medical faculties for comparison.

#### 2.2.2 Lifetime History of NCDs

Participants were asked to self‐report a lifetime history of cardiovascular disease (coronary heart disease, cerebrovascular disease, heart rhythm disorders and others, including heart failure, heart transplant and heart murmurs), chronic respiratory disease (asthma, allergic rhinitis, chronic obstructive pulmonary disease [COPD] and emphysema), diabetes (Type 1 and Type 2), cancer (skin, colorectal, leukaemia, lymphoma, cancer of the female organs and non‐specified) and CMDs (depression, anxiety, eating and other psychiatric disorders, Alzheimer's disease and dementia).

#### 2.2.3 Metabolic Factors

Body mass index (BMI) was calculated from the participants’ self‐reported weight and height. The recommended range for a normal BMI is between 18.5 and 24.9 kg/m^2^ [31]. Thus, self‐reported BMI ≥25.0 kg/m^2^ was classified as raised. Self‐reported high blood pressure and high cholesterol status (further referred to as hypertension and dyslipidaemia) were assessed by asking participants whether high blood pressure and/or high cholesterol were past or current conditions.

#### 2.2.4 Lifestyle Factors

Self‐reported non‐specific psychological distress within the last 30 days was assessed using the 10‐item Kessler Psychological Distress Scale (K10). This screening tool is designed to detect non‐specific symptoms of psychological distress relating to symptoms of anxiety and depression and has been validated for use within the South African context (Cronbach's alpha = 0.84) [32]. Items are scored on a 5‐point Likert scale and then added together, giving a score between 10 and 50. This score can be used as a continuous variable, but specific cut‐off values are debated. However, scores of 16–29 and 30–50 have been suggested to indicate probable mild–moderate or serious mental illness, respectively [33]. Alternatively, this score may be further divided into four categories: low (10–15), moderate (16–21), high (22–29) and very high (30–50) [33]. Categorical scoring was used in this study.

Self‐reported physical activity levels were assessed using the International Physical Activity Questionnaire Short Form (IPAQ‐SF), which measures the amount of vigorous, moderate and light physical activity and hours of sedentary time over a 7‐day period [34]. Continuous and categorical scores were analysed, and participants were placed into high, moderate or low categories of physical activity according to the IPAQ scoring manual guidelines. Low levels of physical activity (less than <150 min of moderate activity or <75 min of vigorous activity per week) were classified as inadequate physical activity. Sitting time was assessed through a single question: ‘During the last 7 days, how much time in total did you usually spend sitting on a weekday, per day? (e.g. sitting at a desk, visiting friends, reading, travelling on a bus or sitting or lying down to watch television)’. The IPAQ‐SF has been previously used to determine physical activity and sedentary behaviour among university students [35, 36]. Self‐reported sleep quality was determined on the basis of the Pittsburgh Sleep Quality Index (PSQI), which assesses seven different components of sleep over a 30‐day period. Component scores were combined to provide a summative score between 0 and 21, where lower scores indicate better sleep quality [37]. Self‐reported nutrition (fruit and vegetable and fast‐food consumption) and substance use (frequency and quantity of alcohol use, smoking, and illicit drug use) were assessed using single categorical questions with Likert‐scale responses.

Metabolic‐ and lifestyle factors were dichotomised into the presence of risk factors, coded as ‘1’, or if not present as ‘0’. See Table S1 for the risk factor criteria and examples of associated adverse health effects.

### 2.3 Statistical Analysis

Data were described using frequencies (*n*) and percentages (%) for categorical variables. For continuous variables, the median (25th–75th percentiles) was used. Distribution assumptions for continuous variables were assessed using Kolmogorov–Smirnov and the Shapiro–Wilk tests. Bivariate analyses and comparisons regarding age, level and faculty of study, lifetime prevalence of NCDs and metabolic and lifestyle risk factors were performed using chi‐square tests for categorical variables and Kruskal–Wallis tests for continuous variables, according to men and women. Due to the small sample size of participants identifying as gender‐fluid, these were not included in the further analyses.

LCA was used to determine the participants’ latent risk profiles based on their self‐reported lifetime prevalence of NCDs and metabolic and lifestyle risk factors. Data were assumed to be missing at random (MAR) (see Table S2). Aike's information criteria (AIC), Bayesian information criteria (BIC), entropy and the interpretability of the findings were used to determine which model best suited the data and research aims [38, 39]. Finally, once the model was selected, the item‐response probability (RP) of each variable was assessed for within‐class homogeneity and differentiation between classes [40]. Chi‐square analysis was done to investigate the associations between demographic variables and the identified latent classes. If a significant association was found, further post hoc analyses were done with Bonferroni‐adjusted *p* values to indicate which classes differ significantly. Cramer's *V* correlation coefficient was used as a measure of effect size (>0.25 = very strong; >0.15 = strong; >0.10 = moderate; >0.05 = weak and >0 = no effect) [41].

IBM SPSS Statistics 29.0 was used for the descriptive statistical analysis, and STATA 18 (StataCorp. 2023. Stata Statistical Software: Release 18. College Station, TX: StataCorp LLC.) was used for the LCA. Statistical significance was set at *p* value <0.001 to provide stronger statistical evidence, unless otherwise specified.

### 2.4 Missing Data Handling

LCA is a form of structural equation modelling (SEM) that identifies latent unobserved subgroups within a population based on patterns of responses to indicator variables. Given the missingness patterns and distributions reported for BMI (see Table S2), which had the highest percentage of missingness, the MAR assumption was plausible, and advanced statistical methods consistent, efficient and unbiased under this assumption were implemented. Full Information Maximum Likelihood (FIML) is a powerful and recommended method for handling missing data in SEM modelling, which estimates parameters by maximising the likelihood function using all available data. FIML has been shown to produce unbiased parameter estimates and standard errors under both MAR and Missing Completely at Random (MCAR) [42].

## 3 Results

### 3.1 Participants

A total of 3237 students were included. More than half of the sample were women (59.6%), followed by men (39.1%) and gender‐fluid participants (1.3%) (see Table 1). Most participants were undergraduates (76.5%) from non‐medical faculties (82.2%), with a median age of 21 years (18–66 years). Gender distribution by age and year of study was similar (*p* = 0.504 and 0.723, respectively) but significantly differed by faculty of study (*p* < 0.001).

**TABLE 1:** The participants’ self‐reported demographic characteristics according to men, women and gender‐fluid groups.

		Gender groups				
Characteristics	All	Men	Women	Gender‐fluid		
***n*, %**	3237 (100%)	1266 (39.1%)	1930 (59.6%)	41 (1.3%)	***p***	**MD (%)**
**Age**						106 (3.3)
Median (25th–75th)	21 (19–23)	21 (19–23)	21 (19–23)	20 (19–22.5)	0.504	
Age categories, *n*%					0.681	
<21	1514 (48.4%)	583 (47.5%)	910 (48.9%)	21 (51.2%)		
≥21	1617 (51.6%)	646(52.6%)	951 (51.1%)	20 (48.8%)		
**Year of study**					0.174	12 (0.4)
Undergraduate, *n*%	2475 (76.5%)	971 (76.7%)	1466 (76.0%)	38 (92.7%)		
Postgraduate, *n*%	750 (23.2%)	290 (22.9%)	457 (23.7%)	3 (7.3%)		
**Faculty of study**					**<0.001** ^*^	0
Non‐medical, *n*%	2661 (82.2%)	1111 (87.8%)	1512 (78.3%)	38 (92.7%)		
Medical, *n*%	576 (17.8%)	155 (12.2%)	418 (21.7%)	3 (7.3%)		

*Note:* Continuous data analysed by Mann–Whitney *U* test and categorical analysis done with Pearson chi‐square tests.

Abbreviation: MD, missing data.

* Bold values Statistically significant difference between gender groups with *p* value <0.001.

### 3.2 Lifetime Prevalence of NCDs

A total of 1679 participants (52.5%) reported a lifetime history of at least one NCD, whereas 15.6% reported both an NCD4 and CMD (see Table 2). Chronic respiratory diseases were the most prevalent (34.7%), of which allergic rhinitis (29.2%) and asthma (15.1%) were most common. Self‐reported CMDs were prevalent (31.2%), with depression (24.8%) and anxiety disorders (21.6%) being the most reported. Cardiovascular conditions (2.5%), diabetes (0.8%) and cancer (0.7%) were less common. Women had a significantly higher prevalence of NCDs overall (*p* < 0.001), chronic respiratory conditions overall (*p* < 0.001), allergic rhinitis (*p* < 0.001) and CMDs (*p* < 0.001).

**TABLE 2:** The lifetime prevalence of self‐reported NCDs in men and women.

	Gender groups				
Characteristics	All	Men	Women	*χ* ^2^ (*p*)	MD
*n* (%)	3196 (100)	1266 (39.6)	1930 (60.4)		
**Overall NCD prevalence, *n*(%)**	1679 (52.5)	517 (30.8)	1162 (69.2)	**<0.001** ^*^	0
**Chronic respiratory disease combined, *n*(%)**	1108 (34.7)	368 (29.1)	740 (38.3)	**<0.001** ^*^	0
Asthma	481 (15.1)	179 (14.1)	302 (15.6)	0.243	
Allergic rhinitis	934 (29.2)	294 (23.2)	640 (33.2)	**<0.001** ^*^	
COPD	3 (0.1)	0 (0.0)	3 (0.2)	0.160	
Emphysema	2 (0.1)	1 (0.1)	1 (0.1)	0.764	
**CMDs combined, *n* (%)**	997 (31.2)	238 (18.8)	759 (39.3)	**<0.001** ^*^	0
Anxiety disorder	689 (21.6)	129 (10.2)	560 (29.0)	**<0.001** ^*^	
Depression	794 (24.8)	207 (16.4)	587 (30.4)	**<0.001** ^*^	
Eating disorders	308 (9.6)	25 (2.0)	283 (14.7)	**<0.001** ^*^	
Other psychiatric disorders	127 (4.0)	33 (2.6)	94 (4.9)	0.001	
**Cardiovascular diseases combined, *n* (%)**	81 (2.5)	22 (1.7)	59 (3.1)	0.020	0
Coronary heart disease	18 (0.6)	8 (0.6)	10 (0.5)	0.674	
Cerebrovascular disease	9 (0.3)	1 (0.1)	8 (0.4)	0.080	
Disorders of rhythm	40 (1.3)	7 (0.6)	33 (1.7)	0.004	
Other	25 (0.8)	11 (0.9)	14 (0.7)	0.652	
**Diabetes combined, *n* (%)**	25 (0.8)	13 (1.0)	12 (0.6)	0.201	0
Diabetes Type 1	14 (0.4)	10 (0.8)	4 (0.2)	0.015	
Diabetes Type 2	12 (0.4)	4 (0.3)	8 (0.4)	0.656	
**Cancer combined, *n* (%)**	21 (0.7)	6 (0.5)	15 (0.8)	0.299	0
Skin cancer	5 (0.2)	2 (0.2)	3 (0.2)	0.986	
Colorectal cancer	2 (0.1)	0 (0.0)	2 (0.1)	0.252	
Leukaemia	3 (0.1)	1 (0.1)	2 (0.1)	0.824	
Lymphoma	2 (0.1)	1 (0.1)	1 (0.1)	0.764	
Cancer of female organs	3 (0.1)	0 (0.0)	3 (0.2)		
Other (non‐specified)	6 (0.2)	2 (0.2)	4 (0.2)	0.753	
**NCD4 and CMDs, *n* (%)**	498 (15.6)	115 (9.1)	383 (19.8)	**<0.001** ^*^	0

*Note:* Variables are ranked according to prevalence.

Abbreviations: CMD, common mental disorder; COPD, chronic obstructive pulmonary disorder; MD, missing data; NCD, non‐communicable diseases; NCD4, collective term referring to cardiovascular diseases chronic respiratory diseases, cancer and diabetes.

* Statistically significant difference between gender groups with *p* value <0.001 (bold) highlighted in grey.

### 3.3 Prevalence of Metabolic and Lifestyle Risk Factors

#### 3.3.1 Metabolic Risk Factors

A self‐reported BMI above 25 kg/m^2^ (35.4%) was common, but dyslipidaemia and hypertension had lower but similar prevalence rates of 3.3% and 3.0%, respectively (see Table 3).

**TABLE 3:** The prevalence of self‐reported metabolic and lifestyle risk factors associated with NCD development in men and women.

		Gender group			
Characteristics	All	Men	Women	*χ* ^2^ (*p*)	MD
***n* (%)**	3196 (100)	1266 (39.6)	1930 (60.4)		
**Metabolic risk factors, *n* (%)**					
Raised BMI	763 (35.4)	325 (37.0)	438 (34.3)	0.191	1040 (32.5)
Dyslipidaemia	105 (3.3)	35 (2.8)	70 (3.6)	0.181	
Hypertension	95 (3.0)	43 (3.4)	52 (2.7)	0.253	
**Lifestyle risk factors, *n* (%)**					
Inadequate fruit and vegetable consumption	2908 (91.3)	1172 (93.0)	1736 (90.2)	0.006	11 (0.3)
Any alcohol use	2511 (78.8)	1042 (82.6)	1469 (76.4)	**<0.001** ^*^	10 (0.3)
Excessive sedentary behaviour	1784 (56.3)	678 (54.4)	1106 (57.5)	0.084	53 (1.7)
Poor sleep quality	1697 (54.7)	551 (44.9)	1146 (61.1)	**<0.001** ^*^	92 (2.9)
High levels of psychological distress	1404 (44.0)	398 (31.5)	1006 (52.2)	**<0.001** ^*^	4 (0.1)
Any smoking	656 (20.6)	331 (26.2)	325 (16.9)	**<0.001** ^*^	12 (0.4)
Binge drinking	359 (14.4)	214 (20.6)	145 (10.0)	**<0.001** ^*^	706 (22.1)
Excessive fast‐food consumption	454 (14.3)	200 (15.9)	254 (13.2)	0.035	17 (0.5)
Inadequate physical activity	260 (8.8)	68 (5.9)	192 (10.7)	**<0.001** ^*^	251 (2.9)
Any illicit drug use	236 (7.4)	110 (8.7)	126 (6.6)	0.023	17 (0.5)

*Note:* Variables are ranked according to prevalence.

Abbreviations: BMI, body mass index; IPAQ‐SF, International Physical Activity Questionnaire; K‐10, 10‐item Kessler Psychological Distress Scale; MD, missing data; NCD, non‐communicable disease; PSQI, Pittsburgh Sleep Quality Index.

* Statistically significant difference between gender groups with *p* value <0.001 (bold), highlighted in grey. Raised BMI—BMI ≥ 25 kg/m^2^; high levels of psychological distress—K10 value ≥22; Inadequate physical activity—<150 min moderate activity or <75 min vigorous activity per week (IPAQ‐SF); Excessive sedentary behaviour—≥8 h of sitting time (IPAQ‐SF); Poor sleep quality—PSQI score ≥6; Inadequate fruit and vegetable consumption—<5 servings per day; Excessive fast‐food consumption—≥2 times per week; Binge drinking—≥5 drinks consumed on a typical drinking day.

#### 3.3.2 Lifestyle Risk Factors

Inadequate fruit and vegetable consumption (91.3%), alcohol use (78.3%), excessive sedentary behaviour (56.3%), poor sleep quality (54.7%), high levels of psychological distress (44.0%), smoking (20.6%), excessive fast‐food consumption (14.3%) and illicit drug use (7.4%) were prevalent behaviours among participants (see Table 3). However, only 8.8% of participants had inadequate physical activity based on the IPAQ scoring criteria. Women were more likely to have higher levels of psychological distress (*p* < 0.001), inadequate physical activity (*p* < 0.001) and poor sleep quality (*p* < 0.001). Men were more likely to use alcohol (*p* < 0.001), binge drink (*p *< 0.001) and smoke (*p *< 0.001).

### 3.4 Latent Class Analysis

Two‐, three‐ and four‐class model solutions were investigated for proper fit. The four‐class model did not converge even after using 50 random starting values. For the three‐class solution, the average posterior probabilities were 0.89, 0.84 and 0.87 for classes 1, 2 and 3, respectively (see Table S3). The robustness of these posterior probabilities is supported by an entropy value >0.6 and high average posterior probabilities ranging from 0.84 to 0.89, suggesting high classification certainty and well‐separated, stable classes for the solution [43]. Furthermore, the use of FIML to handle missing data ensures the sample size remains large, and the parameter estimates stay stable. Additionally, 50 randomly selected starting values for the LCA were used, and the solution converged to the same class probabilities. Thus, the three‐class solution demonstrated acceptable stability and class separation and was selected as the optimal model based on overall fit and interpretability. The RPs for each class's variables are presented in Table 4. To assist with interpretation, defining variables with RP > 0.5 are in bold [40].

**TABLE 4:** Latent class analysis: Probability of latent class membership and self‐reported demographic, NCDs and metabolic and lifestyle risk factor item‐response probabilities within each class.

	Lower risk	Moderate‐risk	Higher risk
	1806 (56.5%)	917 (28.7%)	473 (14.8%)
	**Item‐response probabilities**		
**NCDs**			
Chronic respiratory disease	0.26	0.46	0.41
Cardiovascular disease	0.01	0.05	0.04
Cancer	0.00	0.01	0.01
Diabetes Mellitus	0.01	0.01	0.0
CMDs	0.08^a^, ^b^	**0.58**	**0.59**
**Metabolic risk factors**			
Dyslipidaemia	0.02	0.05	0.03
Hypertension	0.02	0.04	0.03
Raised BMI	0.36	0.42	0.40
**Lifestyle risk factors**			
High levels of psychological distress	0.09^a^, ^b^	**0.80**	**0.70**
Inadequate physical activity	0.28	**0.51**	0.40
Poor sleep	0.16^a^, ^b^	**0.72**	**0.70**
Inadequate fruit and veg consumption	**0.90**	**0.93**	**0.92**
Excessive fast food consumption	0.09	0.13	0.21
Any alcohol use	**0.78**	**0.70**	**0.97**
Binge drinking	0.12	0.07	0.31
Any smoking	0.13^a^	0.02^a^	**0.78**
Any illicit drug use	0.02	0.01	0.37

*Note:* To assist with interpretation, variables that were defining of a class (RP > 0.5) were emphasised in bold.

Abbreviations: BMI, body mass index; CMD, common mental disorders; NCDs, non‐communicable diseases.

^a^ Response probability differs ≥0.5 from the higher risk class.

^b^ Response probability differs ≥0.5 from the moderate‐risk class. Raised BMI—BMI ≥ 25 kg/m^2^; High levels of psychological distress—K10 value ≥22; Inadequate physical activity—<150 min moderate activity or <75 min vigorous activity per week (IPAQ‐SF); Poor sleep quality—PSQI score ≥6; Inadequate fruit and vegetable consumption—<5 servings per day; Excessive fast‐food consumption—≥2 times per week; Binge drinking—≥5 drinks consumed on a typical drinking day.

**FIGURE 1:**
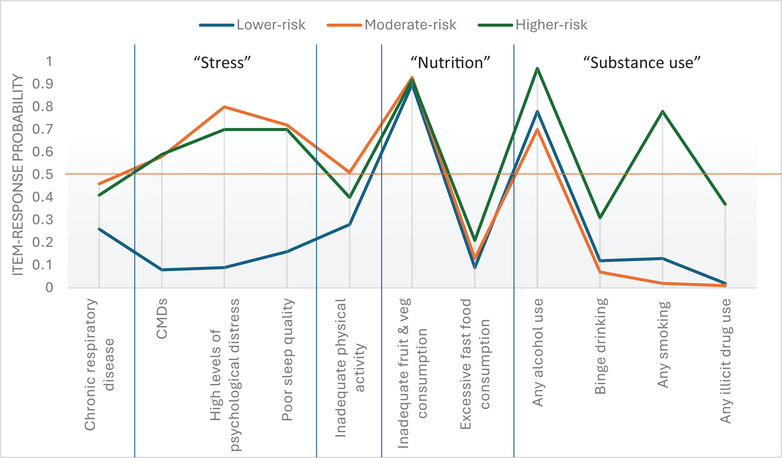
A profile plot of the three‐class latent model depicting the item‐response probabilities of the self‐reported NCD and lifestyle risk factor variables that define or differentiate between the different latent groups. To facilitate interpretation, an orange line on the *Y*‐axis at 0.5 has been added to aid in understanding variables with item‐response probabilities that define classes, and blue lines have been added on the *X*‐axis to delineate the stress‐related (CMDs, high levels of psychological distress and poor sleep quality), nutrition‐related variables (inadequate fruit and vegetable consumption and excessive fast food consumption) and substance use‐related variables (any alcohol use, binge drinking, any smoking and any illicit drug use). High levels of psychological distress—K10 value ≥22; inadequate physical activity—<150 min moderate activity or <75 min vigorous activity per week (IPAQ‐SF); poor sleep quality—PSQI score ≥6. CMD, common mental disorders; NCDs, non‐communicable diseases.

Most participants belonged to Class 1 or the lower risk group (56.5%), who were characterised by a high probability of inadequate fruit and vegetable consumption (RP = 0.90) and any alcohol use (RP = 0.78) (see Table 4). This group was mainly distinguished from Class 2 or moderate‐risk and Class 3 or higher risk by stress‐related factors, such as high levels of psychological distress, CMDs and poor sleep quality, and, to a lesser extent, by chronic respiratory diseases. The moderate‐risk group (28.7%) had similarly high probabilities of CMDs (RP = 0.58 vs. RP = 0.59), high levels of psychological distress (RP = 0.80 vs. RP = 0.70) and poor sleep (RP = 0.72 vs. RP = 0.70) compared to the higher risk group (14.8%) but had the highest probability of inadequate physical activity (RP = 0.51), overall. The higher risk group was further differentiated from the other groups by increased substance use and had the highest probability of engaging in any smoking (RP = 0.78), any illicit drug use (RP = 0.37), binge drinking (RP = 0.31) and any alcohol use (RP = 0.97). A graphical representation of the three‐class model, highlighting the main differentiating variables, is shown in Figure 1.

Certain predictor variables had similar RPs across classes and were not useful for class differentiation (see Table 4). For example, cardiovascular disease, cancer, diabetes, dyslipidaemia and hypertension had low RPs, whereas raised BMI was more common and inadequate fruit and vegetable consumption had high RPs across classes. When the associations between demographic variables and class membership were assessed, only gender was significantly associated with class membership (*p* < 0.001) with a strong effect size (Cramer's *V* = 0.231) (see Table 5). Women were more likely to be in the moderate‐risk (77.6%), followed by the higher risk (59.8%), whereas men were more likely to be in the lower risk group (48.2%) and higher risk group (40.2%). Level and faculty of study were associated with class membership (*p* < 0.05) with weak effect sizes (Cramer's *V* < 0.10).

**TABLE 5:** Pearson's chi‐square analysis of the association between the identified latent classes and the participants’ self‐reported demographic variables.

Characteristics	Lower risk	Moderate‐risk	Higher risk	*χ* ^2^ (*p*)	Cramer's *V*
***n* (%)**	1806 (56.5)	917 (28.7)	473 (14.8)		
**Age *n* (%)**				0.402	0.024
<21	876 (49.3)	411 (46.7)	215 (47.6)		
≥21	890 (50.7)	470 (53.3)	237 (52.4)		
**Gender *n* (%)**					
Women	935^a^ (51.8)	712^b^ (77.6)	283^c^ (59.8)	**<0.001** ^*^	0.231
Men	871^a^ (48.2)	205^b^ (22.4)	190^c^ (40.2)		
**Level of study, *n* (%)**					
Undergraduate	1367^a^ (75.7)	678^a^ (73.9)	392^b^ (82.9)	0.002	0.052
Postgraduate	433^a^ (24.0)	233^a^ (25.4)	81^b^ (17.1)		
**Faculty of study, *n* (%)**					
Medical	332^a^ (18.4)	182^a^ (19.8)	59^b^ (12.5)	0.002	0.062
Non‐medical	1474^a^ (81.6)	735^a^ (80.2)	414^b^ (87.5)		

*Note:* Each superscript letter depicts a subset of the latent classes whose column proportions do not significantly differ from each other at the 0.05 level.

* Bold Statistically significant difference between classes with *p* value <0.001.

## 4 Discussion

This study aimed to describe the prevalence of NCDs in a sample of South African university students and to identify the NCD, metabolic and lifestyle risk factor profiles present that may be targeted for intervention. More than half (52.5%) of participants reported having at least one NCD, of which allergic rhinitis was most common, and a high prevalence of lifestyle risk factors, especially inadequate fruit and vegetable consumption and alcohol use, was present. LCA identified three distinct risk profiles. Stress‐related factors differentiated the moderate‐risk and higher risk groups from the lower risk group, whereas increased substance use further characterised the higher risk group. Gender was the only demographic variable found to significantly influence these findings.

The limited research on NCDs among university students has found prevalences ranging from 16.5% to 29.9% [13, 44, 45, 46, 47]. However, the heterogeneity among studies regarding the NCDs assessed makes comparisons of overall prevalences difficult. One study reported on 13 categories of NCDs, another on only 4, and others were unclear as to which specific NCDs were assessed [13, 45, 46, 47]. When comparing individual NCD prevalences, other university‐based studies also found that asthma (4.2%–26.1%) and allergic rhinitis (6.4%) were the most commonly reported NCDs [13, 48]. The high prevalence of asthma (15.1%) and allergic rhinitis (29.2%) found in this study echoes concerns of a high and increasing prevalence of allergy‐related diseases in sub‐Saharan Africa and warrants further research [49, 50, 51].

Allergy‐related conditions have been found to reduce quality of life, sleep quality and academic performance and have been associated with anxiety and depressive symptoms, which are also prevalent in this study [52, 53, 54, 55, 56]. It is hypothesised that the allergy‐CMD link may be due to the effect of allergy‐related inflammation on the brain's neurotransmitters or indirectly from allergy‐related sleep disturbances that disrupt mood regulation [52, 57]. Despite allergic rhinitis being common, this study's CMD prevalence was lower than that reported in a recent national survey of South African university students (31.2% vs. 53.3%) [58]. However, this discrepancy can be due to the broader spectrum of CMDs included in the national survey. Regardless, the high CMD prevalence in this cohort remains concerning. Furthermore, 15.6% of participants reported having both an NCD4 and a CMD, supporting the notion of an association between these conditions [22].

When comparing this study's prevalence of cardiovascular diseases, diabetes and cancer to other research, cardiovascular conditions and diabetes were more prevalent among American undergraduates (4.2% vs. 2.5%) and United Arab Emirates students (4.2% vs. 0.8%), respectively [45, 48]. Yet, diabetes (0.6% vs. 0.8%) and cancer (0.2% vs. 0.7%) prevalence rates were similar among undergraduates from Serbia, another developing country [13]. This emphasises that NCD trends may differ according to national income [59]. Moreover, when the prevalence of these NCDs was compared to another study done among South African adults (aged 18–35 years), a lower prevalence of coronary artery disease (0.6% vs. 5.8%), cerebrovascular disease (0.3% vs. 1.1%), diabetes (0.8% vs. 7.2%) and cancer (0.7% vs. 1.1%) was observed [24]. As this study relied on self‐reported data, this may suggest that these conditions are underdiagnosed in this population, highlighting the potential to develop interventions to prevent and detect NCD4, given the high prevalence of NCD‐associated risk factors observed.

Compared with a recent South African systematic review, this study found a higher prevalence of inadequate fruit and vegetable consumption (91.3% vs. 44.8%), alcohol use (78.8% vs. 54.8%) and smoking (20.6% vs. 17.9%) but a lower prevalence of inadequate physical activity (8.8% vs. 34.8%–39.0%) and binge drinking (14.4% vs. 31.3%) among its participants [60]. The low prevalence of inadequate physical activity but high alcohol use may be explained by the geographical location of the main campus environment, which is conducive to exercise but also supports a strong drinking culture. In fact, higher levels of binge drinking were anticipated. The prevalence of hypertension and dyslipidaemia was also lower than expected, especially when compared to other South African young adults (3.0% vs. 15.2% and 3.3% vs. 11.2%, respectively) [24]. However, little is known regarding the prevalence of hypertension and dyslipidaemia among South African university students in general. Internationally, the prevalence of hypertension (including pre‐hypertension) and dyslipidaemia among university students varies considerably, ranging from 2.2% to over 40% and 11.2% to 76.5%, respectively [13, 61, 62, 63, 64]. It has been cautioned that self‐reported dyslipidaemia and hypertension are commonly underreported when compared to objectively measured data [65, 66]. Considering this and the co‐occurring high prevalence of raised BMI (34.7%), which has been linked to these conditions, hypertension and dyslipidaemia may also be underdiagnosed in this population [67].

Specific gender differences were noted in lifestyle but not metabolic risk factor patterns. Men were more likely to consume alcohol, binge drink, smoke and have poor nutrition, whereas women were more likely to have high levels of psychological distress, inadequate physical activity and poor sleep quality [60, 68, 69, 70]. Gender differences in health behaviour have been attributed to women being more aware of the benefits of healthy behaviour (except for exercise) and disease prevention [69, 71].

Lastly, LCA identified that the moderate‐ and higher risk classes were characterised less by traditional metabolic risk factors or other NCDs and more by a distinct pattern of stress‐related factors like high levels of psychological distress, poor sleep quality and CMDs. This pattern aligns with the demographic profile of a predominantly young cohort where cardiometabolic diseases, cancer and diabetes are less common [13, 48]. In contrast, stress, CMDs and disrupted sleep are consistently reported among university students navigating this transitional life phase [72, 73, 74, 75]. This study's findings suggest that their current health risk is primarily driven by psychosocial and behavioural pathways rather than by metabolic risk factors or established disease. This cluster of stress‐related variables is also not surprising. Lund et al. showed that stress explained most of the variance in poor sleep, which was associated with self‐reported negative moods and increased alcohol, prescription and recreational drug use [76]. Furthermore, longitudinal studies have demonstrated bidirectional relationships between poor sleep and poor mental health among university students [77, 78, 79]. This may be due to complex neuroendocrine pathways that interfere with the hypothalamic‐pituitary axis and glucocorticoid secretion [76, 80, 81]. Additionally, the stressful nature of university life, with heavy workloads, noisy environments and increased autonomy and independence, may contribute to poor sleep hygiene [76, 82]. Although causality cannot be implied, allergy‐related disorders, inadequate physical activity and substance use, which are more prominent in the higher risk group, have also been associated with poorer sleep and mental health [52, 53, 83, 84].

Despite considerable heterogeneity in risk factors and measurement approaches, certain behavioural patterns can be observed among these participants, similar to those reported in other LCA studies across different national contexts. For example, poor diet, particularly inadequate fruit and vegetable intake, is common across most profiles, whereas higher risk latent groups are mainly distinguished by increased substance use (alcohol use in isolation or in combination with smoking and/or illicit drug use) [2, 3, 27, 28, 85, 86, 87, 88]. Increased stress and poorer mental health outcomes have also been linked with higher risk groups, except for Nazar et al., who reported no difference between groups [2, 3, 85, 87]. Notably, these studies examined mental health variables as external correlates of latent class membership rather than including them as class‐defining indicators, as in this study.

However, our study differed from several previous LCA studies in the observed gender distributions among classes. When the sample's overall gender distribution (women = 60.4%; men = 39.4%) was taken into account, men were overrepresented in the lower risk group (48.2%) and markedly under‐represented in the moderate‐risk group (22.4%), which was characterised by increased psychological distress, poor sleep and CMDs. Contrary to what is often observed, men were not overrepresented in the higher risk group (40.2%), whose gender distribution closely reflected that of the overall sample [3, 28, 85, 87]. This suggests that men were less likely to cluster into predominantly stress‐related profiles but were equally represented in the higher risk profile when stress‐related factors co‐occurred with increased substance use.

Collectively, these findings support a dual prevention approach, combining population‐level strategies to address the pervasiveness of poor dietary habits with targeted, gender‐sensitive, multi‐behavioural interventions for higher risk groups characterised by co‐occurring stress‐related factors and increased substance use.

## 5 Study Limitations

Like other questionnaire‐based studies, this study is limited by reliance on self‐reported data, which can be influenced by recall and social desirability biases [9]. As mentioned, self‐reported hypertension and dyslipidaemia data should be used with caution [65, 66]. BMI accuracy has also been questioned, as men tend to overreport their height and women tend to underreport their weight [89]. Furthermore, self‐reported mental health diagnoses can lead to misclassification or under‐reporting, especially when describing specific subgroups of CMDs [90]. However, self‐reported diagnoses of commonly known CMDs, such as depression, correlate well with symptom‐based diagnoses and can be used as broad clinical indicators [70, 71]. It has also been suggested that self‐reported CMDs among college students are more accurate than those in a general adult population due to reduced stigma and increased mental health awareness [91]. Future studies in this cohort will benefit from the confirmation of the self‐reported conditions, anthropometric measurements and physical activity levels by a medical practitioner.

A limitation of the MHP is that it did not include other sociodemographic factors, such as household income. This could influence participants’ health profile and risk factors, as low socioeconomic status has been associated with risk factor behaviour [92]. Additionally, due to the limited number of participants, gender‐fluid individuals were excluded from further analysis. As LCA assumes conditional independence, it implies that latent class membership explains all the associations among the observed items, which is another limitation. However, the tenability of this assumption could not be assessed because evaluation methods are better designed for continuous variables rather than the binary variables used in this study.

Final limitations include the inability to determine a precise response rate due to incomplete data collection metrics and the limited representativeness of the broader university population. Student participation in completing the MHP was promoted through awareness drives and university newsletters. According to Everlytic, a large‐scale communication software platform, 55,726 emails with MHP links were delivered to university staff and students in 2021, with 19,671 opened (35.3%). In 2022, 36,872 emails were delivered, with 19,063 opened (51.7%). This demonstrates that the MHP was made available to many university students to be inclusive. Still, the findings of this study are not generalisable and may be affected by non‐response bias [93]. Non‐response bias has been associated with certain participant demographics, such as younger men, unhealthy behaviours and even higher mortality rates [93, 94, 95, 96]. However, in general, non‐response bias is not solely responsible for lower response rates, which are also influenced by age, education, socioeconomic status, ethnicity and topic interest [93, 97]. Considering this sample's demographics, topic interest is likely to have influenced the response rate, with health‐oriented individuals being more likely to respond [93]. As a result, this study's findings may be biased toward a healthier, health‐conscious subgroup, leading to an underestimation of NCD and NCD‐associated risk factor prevalence in the broader student population. Furthermore, selection bias may have occurred because only online recruitment via a mailer was conducted, potentially affecting students with less reliable internet access [98]. Future studies can consider alternative recruitment methods and sending additional reminders to complete the MHP, which has been found to increase response rates. However, this approach appears to have little effect on study outcomes [96, 99].

## 6 Study Implications

To our knowledge, this study is the first to determine NCD and NCD‐associated latent risk profiles among South African university students and to directly integrate NCDs into an LCA. Our findings demonstrate that university students represent an ‘early‐risk’ population with health risk driven primarily by stress‐related and behavioural factors rather than established cardiometabolic disease.

Although health promotion initiatives are implemented in this setting, guided by the South African National Health Awareness Calendar, these efforts remain broad. In a low‐resource context, these findings highlight the value of online surveillance tools, such as the MHP, and advanced statistical techniques, such as LCA, for determining risk profiles and informing more efficient, targeted prevention strategies.

Aligned with WHO recommendations, these findings support earlier, integrated NCD prevention strategies that address CMDs and other NCDs simultaneously, particularly among young adults, as CMDs were key components of higher risk profiles [21]. Rather than prioritising less common cardiometabolic diseases, cancer or diabetes, targeted intervention efforts should focus on identifying and addressing stress‐related factors and increased substance use, using scalable, low‐cost interventions delivered through peer‐based models, digital platforms and targeted health messaging. Intervention design should incorporate gender‐sensitive engagement and detection strategies, as reliance on stress‐related factors may preferentially identify women, whereas under‐detecting higher risk patterns in men. Finally, at the university governance level, the findings support shifting from awareness‐based initiatives toward system‐level action to create health‐enabling environments, as outlined by the Okanagan Charter [5]. This may include regulating residential noise, adjusting food and alcohol costs and availability and providing general health education on sleep and substance use.

## 7 Conclusion

This study provides novel evidence on the burden and risk profiles of NCDs, metabolic and lifestyle risk factors among South African university students. By combining an online surveillance tool with LCA‐based risk profiling, this study offers a cost‐effective, scalable approach to identify and support higher risk groups in low‐resource settings. Within this context, although cardiovascular diseases, diabetes and cancer were less common, more than half of participants reported at least one NCD, with allergy‐related chronic diseases and CMDs being most prevalent. LCA identified that poor diet was common across all profiles, and three distinct risk profiles were identified, with the higher risk group characterised primarily by stress‐related factors, such as psychological distress, poor sleep and CMDs and increased substance use.

As the Okanagan Charter suggests, universities are critical settings for early NCD prevention, and these findings position university students as an ‘early‐risk’ population that requires integrated prevention approaches. In this setting, potential prevention strategies may include increased screening for stress‐related factors and alcohol use at the individual level, population‐level sleep hygiene and stress management programmes and targeted support for students with co‐occurring risks (higher risk groups) through low‐cost digital or peer‐based models. Additionally, gender‐sensitive approaches are needed to avoid under‐detection of higher‐risk patterns, particularly among men. Finally, at the institutional level, universities should consider revising their food, alcohol and noise policies on campus to promote healthier environments. Future research should examine longitudinal transitions among risk profiles, validate self‐reported health measures against objective measures and evaluate the effectiveness of risk‐profile‐informed interventions in university settings.

## Author Contributions

**Melissa Janse van Vuren**: concept and design, data cleaning and statistical analysis, drafting of the manuscript. **Jason Bantjes**: concept and design, contributions toward data interpretation, critical revision of manuscript. **Liske Kotze‐Horstmann**: concept and design, contributions toward data interpretation, critical revision of manuscript. **Innocent Maposa**: data cleaning and statistical analysis, critical revision of manuscript. **Wayne Derman**: contributions toward data interpretation, critical revision of manuscript.

## Funding

The work reported herein was made possible through funding by the South African Medical Research Council through its Division of Research Capacity Development under the SAMRC Institutional Clinician Researcher Development Programme with funding received from the National Department of Health.

## Ethics Statement

Ethical approval for this study was obtained from the Health Research Ethics Committee of Stellenbosch University (Project ID: 30036; HREC Reference No: S24/02/047) in alignment with the recommendations of the Declaration of Helsinki.

## Consent

Electronic informed consent was obtained from all participants included in this study for the use of their de‐identified data for research purposes.

## Conflicts of Interest

The authors declare no conflicts of interest.

## Supporting information




**Table S1**: An overview of the NCD‐associated risk factor variables’ definitions/criteria used in this study, with examples of associated adverse health effects.

**Table S2**: Assessment of systemic missingness in BMI.

**Table S3**: Overview of criteria to assess model fit for latent class analysis for model selection.

## Data Availability

The data that support the findings of the current study are available from the corresponding author on reasonable request.
