# Supplementary material for: Non‐Communicable Disease, Metabolic and Lifestyle Risk Factor Profiles in South African University Students: A Latent Class Analysis
Source: Public Health Chall. 2026 Apr 9;5(2):e70221. doi: 10.1002/puh2.70221 (PMC13063396; doi:10.1002/puh2.70221)
Supplement: Supplementary file 1 — Table S1: An overview of the NCD‐associated risk factor variables’ definitions/criteria used in this study, with examples of associated adverse health effects. [file PUH2-5-e70221-s001.docx]

| Supplementary Table 1: An overview of the NCD-associated risk factor variables’ definitions/criteria used in this study, with examples of associated adverse health effects. | | | |
| --- | --- | --- | --- |
| Risk factor | **Screening tool/Calculation** | **Definition/criteria** | **Examples of adverse health effects** |
| High levels of psychological distress | K10 | Participants who had a combined K10 score of ≥ 22.^1^ | High K10 scores have been significantly associated with other CMDs.^2^ |
| High BMI | Calculated from self-reported height and weight obtained in MHP | BMI that was calculated to be ≥25.0 kg/m2 according to the WHO criteria.^3^ | Being overweight has been associated with increased risk for cardiovascular disease, diabetes and cancer and can negatively impact your quality of life.^3^ |
| Hypertension | Single question in MHP | Participants who selected that high blood pressure was either a past or current condition. | Hypertension has been associated with increased risk for various cardiovascular and renal conditions.^4^ |
| Dyslipidaemia | Single question in MHP | Participants who selected that high cholesterol was either a past or current condition. | Dyslipidaemia has been associated with increased risk of cardiovascular disease and stroke.^5^ |
| Inadequate physical activity | IPAQ-SF | Participants who did not meet IPAQ-SF “moderate” or “high” levels of physical activity and were less than the recommended minimum requirement of physical activity by WHO guidelines (< 150 minutes of moderate or 75 minutes of vigorous activity or an equivalent combination throughout the week).^6^ | Physical inactivity is associated with an increased risk of obesity, cardiovascular disease, cancer, diabetes and poor mental health.^6^ |
| Excessive sedentary behaviour | IPAQ-SF | A self-reported sitting time exceeding eight hours per day.^7,8^ | Excessive sedentary behaviour has been associated with increased risk for cardiovascular disease and higher all-cause mortality rates.^7,9^ |
| Poor sleep quality | PSQI | A global Pittsburgh Sleep Quality Index (PSQI) score of ≥ 6.^10^ | A score of ≥ 6 has been associated with poor sleep quality, sleep disorders and poor mental health.^11,12^ |
| Inadequate fruit and vegetable consumption | Single question in MHP | Participants consuming less than five portions of fruits and vegetables per day.^13^ | Poor nutrition has been associated with increased risk for diabetes, cardiovascular disease, strokes and cancer.^13^ |
| Excessive fast-food consumption | Single question in MHP | Participants who consume fast food/takeaways two or more times a week.^14^ | Excessive fast food consumption has been associated with increased risk for obesity, poor nutrition and insulin resistance.^15,16^ |
| Any alcohol use | Single question in MHP | Participants who reported any use of alcohol. | Alcohol use has been associated with increased prevalence of NCDs (cancer in particular) and CMDs and premature mortality and disability.^17,18^ |
| Binge drinking | Single question in MHP | Participants who reported the consumption ≥5 standard drinks per occasion (Centre for Disease Control guidelines suggest ≥4 drinks per occasion to be binge drinking in females; however, due to the MHP structure, a cut-off of ≥5 drinks were used for both gender groups).^19^ | Binge drinking has been associated with increased violence, injuries, sexually transmitted infections, alcohol poisoning and overdose.^20^ |
| Any smoking | Single question in MHP | Participants who reported any smoking. | Direct and indirect tobacco use has been associated with cardiovascular and respiratory disease and various cancers.^21^ |
| Any illicit drug use | Single question in MHP | Participants who reported any use of illicit substances | Illicit drug use has been associated with negative socioeconomic effects and increased risk of contracting infection (intravenous drug use).^22^ |
| NCD - non-communicable disease; MHP – MaRooN Health Passport; CMD – common mental disorder; WHO – World Health Organisation; BMI – body mass index; K-10 -10-item Kessler Psychological Distress Scale; IPAQ-SF – International Physical Activity Questionnaire; PSQI - Pittsburgh Sleep Quality Index. | | | |

# References

[1] Hutchesson MJ, Duncan MJ, Oftedal S, Ashton LM, Oldmeadow C, Kay-Lambkin F, et al. Latent Class Analysis of Multiple Health Risk Behaviors among Australian University Students and Associations with Psychological Distress. *Nutrients*. 2021;13(2):425. doi:10.3390/nu13020425

[2] Andrews G, Slade T. Interpreting scores on the Kessler Psychological Distress Scale (K10). *Aust Nz J Publ Heal*. 2001;25(6):494-497. doi:10.1111/j.1467-842x.2001.tb00310.x

[3] World Health Organization. Obesity and overweight: Fact sheets. Published March 1, 2024. <https://www.who.int/news-room/fact-sheets/detail/obesity-and-overweight>. Accessed February 20, 2025

[4] World Health Organization. Hypertension: Fact sheets. Published March 16, 2023. <https://www.who.int/news-room/fact-sheets/detail/hypertension>. Accessed May 7, 2024

[5] World Health Organization. The Global Health Observatory: Raised cholesterol. Published 2024. <https://www.who.int/data/gho/indicator-metadata-registry/imr-details/3236>. Accessed May 7, 2024

[6] World Health Organization. Physical activity. Fact sheets. Published October 5, 2022. <https://www.who.int/news-room/fact-sheets/detail/physical-activity>. Accessed May 7, 2024

[7] Patterson R, McNamara E, Tainio M, Sá TH de, Smith AD, Sharp SJ, et al. Sedentary behaviour and risk of all-cause, cardiovascular and cancer mortality, and incident type 2 diabetes: a systematic review and dose response meta-analysis. *Eur J Epidemiology*. 2018;33(9):811-829. doi:10.1007/s10654-018-0380-1

[8] Edelmann D, Pfirrmann D, Heller S, Dietz P, Reichel JL, Werner AM, et al. Physical Activity and Sedentary Behavior in University Students–The Role of Gender, Age, Field of Study, Targeted Degree, and Study Semester. *Front Public Heal*. 2022;10:821703. doi:10.3389/fpubh.2022.821703

[9] Chau JY, Grunseit AC, Chey T, Stamatakis E, Brown WJ, Matthews CE, et al. Daily Sitting Time and All-Cause Mortality: A Meta-Analysis. *PLoS ONE*. 2013;8(11):e80000. doi:10.1371/journal.pone.0080000

[10] Buysse DJ, Reynolds CF, Monk TH, Berman SR, Kupfer DJ. The Pittsburgh sleep quality index: A new instrument for psychiatric practice and research. *Psychiat Res*. 1989;28(2):193-213. doi:10.1016/0165-1781(89)90047-4

[11] Buysse DJ, Hall ML, Strollo PJ, Kamarck TW, Owens J, Lee L, et al. Relationships between the Pittsburgh Sleep Quality Index (PSQI), Epworth Sleepiness Scale (ESS), and clinical/polysomnographic measures in a community sample. *J Clin sleep Med : JCSM : Off Publ Am Acad Sleep Med*. 2008;4(6):563-571.

[12] Aloba OO, Adewuya AO, Ola BA, Mapayi BM. Validity of the Pittsburgh Sleep Quality Index (PSQI) among Nigerian university students. *Sleep Med*. 2007;8(3):266-270. doi:10.1016/j.sleep.2006.08.003

[13] World Health Organization. Healthy diet: Fact sheets. Published April 29, 2020. <https://www.who.int/news-room/fact-sheets/detail/healthy-diet>. Accessed May 10, 2024

[14] Odegaard AO, Koh WP, Yuan JM, Gross MD, Pereira MA. Western-Style Fast Food Intake and Cardiometabolic Risk in an Eastern Country. *Circulation*. 2012;126(2):182-188. doi:10.1161/circulationaha.111.084004

[15] Boutelle KN, Fulkerson JA, Neumark-Sztainer D, Story M, French SA. Fast food for family meals: relationships with parent and adolescent food intake, home food availability and weight status. *Public Heal Nutr*. 2006;10(1):16-23. doi:10.1017/s136898000721794x

[16] Pereira MA, Kartashov AI, Ebbeling CB, Horn LV, Slattery ML, Jacobs DR, et al. Fast-food habits, weight gain, and insulin resistance (the CARDIA study): 15-year prospective analysis. *Lancet*. 2005;365(9453):36-42. doi:10.1016/s0140-6736(04)17663-0

[17] World Health Organization. Alcohol. Fact sheets. Published May 9, 2022. <https://www.who.int//news-room/fact-sheets/detail/alcohol/?gad_source=1&gclid=Cj0KCQjwxeyxBhC7ARIsAC7dS3_muZ_h7Xtk8aBxUJvuWgzTxqHbdlEv-WFPLcHIrw7rP77d-WDIt10aAvyJEALw_wcB>. Accessed May 8, 2024

[18] Anderson BO, Berdzuli N, Ilbawi A, Kestel D, Kluge HP, Krech R, et al. Health and cancer risks associated with low levels of alcohol consumption. *Lancet Public Heal*. 2023;8(1):e6-e7. doi:10.1016/s2468-2667(22)00317-6

[19] Centre of Disease Control. Alcohol and Public Health: Data on Excessive Drinking. Published August 6, 2024. <https://www.cdc.gov/alcohol/excessive-drinking-data/?CDC_AAref_Val=https://www.cdc.gov/alcohol/data-stats.htm>. Accessed February 21, 2025

[20] Centre for Disease Control. Alcohol Use: Alcohol Use and Your Health. Published January 31, 2025. <https://www.cdc.gov/alcohol/about-alcohol-use/index.html#cdc_behavioral_basics_types-effects-of-short-term-alcohol-use>. Accessed February 21, 2025

[21] World Health Organization. Tobacco: Fact sheets. Published July 31, 2023. <https://www.who.int/news-room/fact-sheets/detail/tobacco>. Accessed May 8, 2024

[22] World Health Organization. Substance Abuse. Published 2024. <https://www.afro.who.int/health-topics/substance-abuse>. Accessed May 10, 2024
