# Supplementary material for: Non‐Communicable Disease, Metabolic and Lifestyle Risk Factor Profiles in South African University Students: A Latent Class Analysis
Source: Public Health Chall. 2026 Apr 9;5(2):e70221. doi: 10.1002/puh2.70221 (PMC13063396; doi:10.1002/puh2.70221)
Supplement: Supplementary file 2 — Table S2: Assessment of systemic missingness in BMI. [file PUH2-5-e70221-s002.docx]

| **Supplementary Table 2. Assessment of systemic missingness in BMI** | | | | |
| --- | --- | --- | --- | --- |
|  | | **Observed** | **Missing** |  |
| **Characteristics** | | **N=2178** | **N=1018** | **χ^2^ (P)** |
|  | **Gender** |  |  | 0.020 |
|  | Men | 893 (41.0%) | 373 (36.6%) |  |
|  | Women | 1,285 (59.0%) | 645 (63.4%) |  |
|  | **Age** |  |  |  |
|  | Age categories |  |  | <0.001 |
|  | <21 | 1260 (59.9%) | 669 (67.8%) |  |
|  | ≥21 | 844 (40.1%) | 317 (32.2%) |  |
|  | **Year of study** |  |  | <0.001 |
|  | Undergraduate | 1,604 (74.0%) | 833 (82.0%) |  |
|  | Postgraduate | 564 (26.0%) | 183 (18.0%) |  |
|  | **Faculty of study** |  |  | <0.001 |
|  | Non-medical | 1,894 (87.0%) | 729 (71.6%) |  |
|  | Medical | 284 (13.0%) | 289 (28.4%) |  |
|  | **NCDs** |  |  |  |
|  | Chronic respiratory disease | 781 (35.9%) | 327 (32.1%) | 0.042 |
|  | Cardiovascular disease | 55 (2.5%) | 26 (2.6%) | 1.00 |
|  | Cancer | 13 (0.6%) | 8 (0.8%) | 0.64 |
|  | Diabetes Mellitus | 19 (0.9%) | 6 (0.6%) | 0.52 |
|  | CMDs | 712 (32.7%) | 285 (28.0%) | 0.008 |
|  | **Metabolic risk factors** |  |  |  |
|  | Dyslipidaemia | 76 (3.5%) | 29 (2.8%) | 0.39 |
|  | Hypertension | 65 (3.0%) | 30 (2.9%) | 1.00 |
|  |  |  |  |  |
|  | **Lifestyle risk factors** |  |  |  |
|  | High levels of psychological distress | 919 (42.2%) | 376 (36.9%) | 0.005 |
|  | Inadequate physical activity | 196 (9.6%) | 64 ( 7.0%) | <0.001 |
|  | Poor sleep | 887 (41.8%) | 402 (40.9%) | 0.67 |
|  | Inadequate fruit & veg consumption | 1,986 (91.5%) | 922 (90.8%) | 0.54 |
|  | Excessive fast food consumption | 266 (12.3%) | 129 (12.7%) | 0.73 |
|  | Any alcohol use | 1717 (79.1%) | 794 (78.2%) | 0.530 |
|  | Binge drinking | 217 (12.7%%) | 142 (18.1%) | <.001 |
|  | Any smoking | 448 (20.6%) | 208 (20.5%) | 0.960 |
|  | Any illicit drug use | 165 (7.61%) | 71 (7.02%) | 0.556 |
| Data are presented as n (%) for categorical measures. BMI was used to define the pattern of missingness because it had the highest percentage of data missing (32%). High levels of psychological distress – K10 value ≥22; Inadequate physical activity - <150min moderate activity or <75min vigorous activity per week (IPAQ-SF); Poor sleep quality – PSQI score ≥6; Inadequate fruit and vegetable consumption - <5 servings per day; Excessive fast-food consumption - ≥ 2 times per week; Binge drinking - ≥5 drinks consumed on a typical drinking day. P - NCDs – non-communicable diseases; CMD – common mental disorders; BMI – body mass index. | | | | |
