# Supplementary material for: Non‐Communicable Disease, Metabolic and Lifestyle Risk Factor Profiles in South African University Students: A Latent Class Analysis
Source: Public Health Chall. 2026 Apr 9;5(2):e70221. doi: 10.1002/puh2.70221 (PMC13063396; doi:10.1002/puh2.70221)
Supplement: Supplementary file 3 — Table S3: Overview of criteria to assess model fit for latent class analysis for model selection. [file PUH2-5-e70221-s003.docx]

| **Supplementary Table 3. Overview of criteria to assess model fit for latent class analysis for model selection.** | | | | | | | |
| --- | --- | --- | --- | --- | --- | --- | --- |
| **Model** | **LL** | **AIC** | **BIC** | **Entropy** | **Posterior probability margin (95% CI)** | | |
|  |  |  |  |  | 1^st^ Class | 2^nd^ Class | 3^rd^ Class |
| **1** | -20508.97 | 41051.95 | 41155.13 | - | - | - | - |
| **2** | -19726.15 | 39522.30 | 39734.74 | 0.674 | 0.90 (0.89-0.91) | 0.92 (0.91-0.92) | - |
| **3** | -19532.07 | 39170.14 | 39491.84 | 0.690 | 0.89 (0.88-0.89) | 0.84 (0.83-0.85) | 0.87 (0.86-0.89) |
| **4** | -19418.23 (not concave) | - | - | - | - | - | - |
| LL – Log-likelihood; AIC – Akaike Information Criteria; BIC – Bayesian Information Criteria; CI – confidence interval. | | | | | | | |
